# Seeker-Azimuth Determination with Gyro Rotor and Optoelectronic Sensors

**DOI:** 10.3390/s18041256

**Published:** 2018-04-19

**Authors:** Jian-Ming Bai, Guangshe Zhao, Hai-Jun Rong, Xianhua Wang

**Affiliations:** 1 State Key Laboratory for Strength and Vibration of Mechanical Structures, Shaanxi Key Laboratory of Environment and Control for Flight Vehicle, School of Aerospace, Xi’an Jiaotong University, Xi’an 710049, China; bai_jm@stu.xjtu.edu.cn or bjm@opt.ac.cn; 2 Optical Direction and Pointing Technique Research Department, Xi’an Institute of Optics and Precision Mechanics of CAS, Xi’an 710119, China; xhwang@opt.ac.cn; 3 School of Electronic and Information Engineering, Xi’an Jiaotong University, Xi’an 710049, China; zhaogs@mail.xjtu.edu.cn

**Keywords:** seeker azimuth, gyro rotor, optoelectronic sensors, duty ratio

## Abstract

This paper presents an approach to seeker-azimuth determination using the gyro rotor and optoelectronic sensors. In the proposed method, the gyro rotor is designed with a set of black and white right spherical triangle patterns on its surface. Two pairs of optoelectronic sensors are located symmetrically around the gyro rotor. When there is an azimuth, the stripe width covering the black and white patterns changes. The optoelectronic sensors then capture the reflected optical signals from the different black and white pattern stripes on the gyro rotor and produce the duty ratio signal. The functional relationship between the measured duty ratio and the azimuth information is numerically derived, and, based on this relationship, the azimuth is determined from the measured duty ratio. Experimental results show that the proposed approach produces a large azimuth range and high measurement accuracy with the linearity error of less than 0.005.

## 1. Introduction

**Figure 1:**
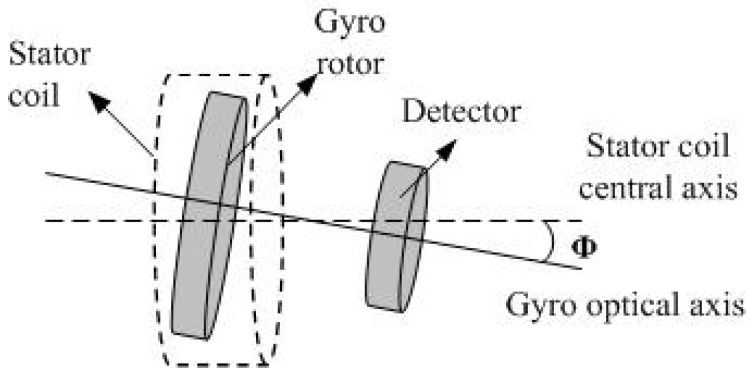
Diagram of the seeker azimuth.

One of the most common problems in navigation and positioning is the determination of the azimuth [1]. Different methods [1,2,3,4,5,6] have been proposed in existing work to determine the azimuth. However, these works have mainly been developed for the measurement of inertial azimuth, which is the angle between the north direction and the projection of the initial plane onto the launch location. This is required in the inertial navigation system and in the initial launch stage. The seeker is an important part of the navigation system and has been used to accurately search and track a target by determining the target position in the field of view [7]. In the seeker [8,9,10,11,12], the input of the system is the space coordination, and its output is the target position, which is expressed by the deviation signal or the correction signal. This system generally consists of an optical gyroscope part and an electronic detector part, as shown in Figure 1. There is an azimuth Φ between the gyro optical axis and the stator coil axis that represents the orientation of the target. Therefore, the azimuth provides the significant signal source for the off-boresight launching and aiming of the follow-up target. As the target is maneuvered or varied, the detector’s output signal changes with the amplitude proportional to the azimuth accordingly. Then, the azimuth is input to the navigation system so that the target is captured accurately. In this case, the azimuth determination and measurement accuracy plays a key role in the navigation system and affects the accuracy of the guidance system.

Compared with the inertial azimuth determination, little work has been done in the determination of the seeker azimuth. The traditional way to measure the seeker azimuth is based on the electromagnetic technique [13]. The electromotive force around the electromagnetic coils is induced when the rotor is rotating the stator and is further transformed into the voltage. The induced voltage has a sinusoidal relation with the azimuth. Using the relationship, the azimuth is obtained. However, the method only has a good performance in measuring the small azimuth angles. Moreover, the electromagnetic environment easily produces magnetic interference. These further affect azimuth measurement accuracy.

Optoelectronic sensors are electronic detectors that convert light, or a change in light, into an electronic signal and they have been used in many applications including azimuth determination [14,15,16,17,18]. A method of obtaining the precise position and azimuth of ground vehicles rapidly based on vehicular bi-axis optical-electronic detector is given in [18]. The system calculates the azimuth of targets relative to the vehicle using the position information provided by the vehicular navigation system. In contrast to the electromagnetic technique, the optoelectronic sensors provide a noncontact measurement method that is effective at avoiding the electromagnetic interference.

Considering this merit, a noncontact approach is proposed to measure the large azimuth angles by detecting pattern information on the surface of gyro rotor based on the optoelectronic sensor. In the proposed approach, the gyro rotor is designed with a set of black and white right spherical triangle patterns on its surface. The optoelectronic sensors are applied to detect the pattern information on the surface of the gyro rotor. In the case of an azimuth, the black and white patterns are changed accordingly. The optoelectronic sensors then capture the reflected optical signals from the varied black and white pattern on the gyro rotor and produce the duty ratio signal. Finally, according to the functional relationship between the measured duty ratio and the azimuth information, the azimuth is determined from the measured duty ratio. This method effectively avoids the electromagnetic interference and achieves large azimuth measurement with high accuracy.

The rest of the paper is organized as follows. In the next section we describe the model of the black and white right spherical triangle patterns on gyro rotor. In Section 3, the duty ratio and the azimuth in the case of one-dimensional rotation is introduced. In Section 4, we describe the duty ratios and the azimuth in the case of two-dimensional rotation. Section 5 gives the numerical solution of the functional relationship between the azimuth and the duty ratios. In Section 6, we describe the experimental results that are obtained when testing the concept. Finally, in Section 7, we present the conclusion of this work.

## 2. Model of Black and White Right Spherical Triangle Graphics on the Gyro Rotor

**Figure 2:**
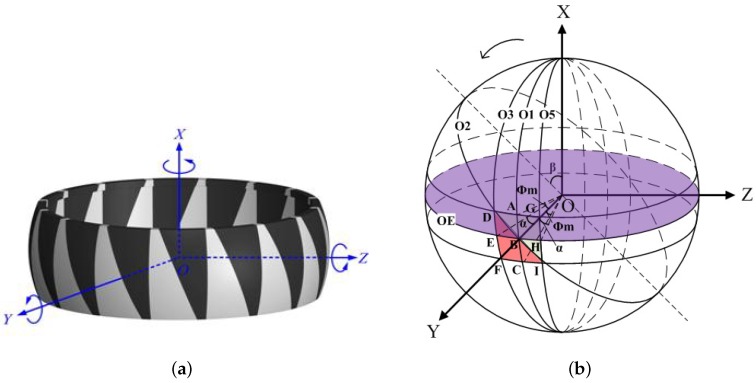
(**a**) Gyro rotor graphics. (**b**) Spherical coordinate OXYZ.

The heart of the new proposed scheme for determining the seeker azimuth is composed (see Figure 2a) of a gyro rotor with a set of black and white right spherical triangle patterns on its surface. The scheme needs to satisfy the design goal as follows: the azimuth is in the range [0, 40], and the linearity error is less than 0.005. The details of the proposed azimuth determination scheme are described in the following.

To describe the mathematical model of the black and white spherical triangular patterns well, a spherical coordinate OXYZ as shown in Figure 2 is constructed on the rotor frame. The fundamental plane is the equinoctial circle O*_E_*. The origin O is the rotor’s center. The X-axis coincides with the inertial axis of the gyro rotor and points upward. The Y-axis coincides with the line connecting the spherical center O and the crossing point B between the right spherical triangle hypotenuse (the arc DBI) and the circle O*_E_*. It is important to keep in mind that the OXYZ system is not fixed and rotated with the rotor. Assuming that the numbers for the black and white triangular patterns are both *n*, the angles α covering the black pattern and the white pattern on the circle O*_E_* are equal, as $\frac{2\pi }{2n}=\frac{\pi }{n}$. The white triangular pattern is marked in red color throughout the paper for clarity. The spherical center angles corresponding to the lower surface and the upper surface of the rotor is expressed as $\phi_{m}$.

As shown in Figure 2b, the circle O_2_ across the arc DBI can be obtained by a β counter-clockwise rotation of the longitudinal circle O_1_ across the arc ABC about OY and expressed as

(1) $$\left\{\begin{array}{c} x^{2}+y^{2}+z^{2}=R^{2}\\ x\sin\beta +z\cos\beta =0 \end{array}\right.$$

where (x,y,z) is the position vector in the OXYZ frame, and *R* is the radius of circle O_1_. Similarly, the circle O_3_ across points D, E, and F can be obtained by an α counter-clockwise rotation of the longitudinal circle O_1_ about OX and expressed as

(2) $$\left\{\begin{array}{c} x^{2}+y^{2}+z^{2}=R^{2}\\ y\sin\alpha +z\cos\alpha =0 \end{array}\right..$$

Here, the radiuses of circles O_1_, O_2_, and O_3_ are equal to *R*. The intersection D between the two circles O_2_ and O_3_ is represented as $\left(R,-\alpha ,\frac{\pi }{2}-\phi_{m}\right)$ under the spherical coordinate system. Based on the relationship between the spherical coordination expression and the rectangular coordinate expression, the point D is further written as

(3) $$\left\{\begin{array}{c} x_{D}=R\cos\left(\frac{\pi }{2}-\phi_{m}\right)\\ y_{D}=R\sin\left(\frac{\pi }{2}-\phi_{m}\right)\cos\alpha \\ Z_{D}=-R\sin\left(\frac{\pi }{2}-\phi_{m}\right)\sin\alpha \end{array}\right..$$

Substituting Equation (3) into Equations (1) and (2) yields

(4) $$\tan\beta =\frac{\sin\alpha }{\tan\phi_{m}}.$$

Equation (4) shows a useful relationship among β, α, and $\phi_{m}$ and depicts the model information of the black and white right spherical triangle patterns on the rotor, which is next used to calculate the azimuth Φ.

In the proposed azimuth determination system, optoelectronic sensors are required to detect the reflected optical signals from the black and white patterns according to the means of rotation. When the rotor has one-dimensional rotation, i.e., rotation about the Y-axis or Z-axis, one optoelectronic detector is required and configured along the rotated Y-axis or Z-axis. Two optoelectronic detectors are necessary for the two-dimensional rotation and placed about the rotated Y-axis and Z-axis. Whatever the rotation type is, the optical spot is consistent to equinoctial circle O*_E_* in the OXYZ frame when the light beam of the optoelectronic is injected on the rotor spinning about its inertial OX-axis. When the azimuth is zero, the stripe width covering the black and white patterns on the circle O*_E_* is equal. Once there is an azimuth, the stripe width changes. Moreover, the reflection rate from the black and white patterns of the rotor is opposite; thus, the reflected optical signals can be transformed to the high-level and low-level pulse signals via the optoelectronic detector, respectively, from which a pulse duty ratio signal K is deduced. In principle, the duty ratio is related with the black and white stripe widths when the rotor rotates at a certain azimuth. Thus, we can extract the azimuth information from the duty ratio signal.

In our study, we mainly consider the case of two-dimensional rotation. Since one-dimensional rotation is the basis of two-dimensional rotation, the duty ratio and azimuth in the one-dimensional rotation are first described in the following. We then enter into the description of the two-dimensional rotation case.

## 3. One-Dimensional Rotation

**Figure 3:**
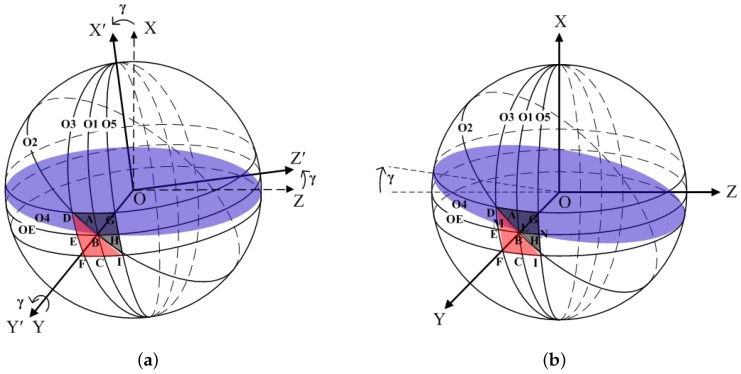
(**a**) Rotation of OXYZ about OY. (**b**) Rotation of circle O_4_ about OY.

In the one-dimensional rotation case, the rotor can rotate about the Y-axis or Z-axis. This corresponds to the pitch attitude or the yaw attitude of the object. However, whatever axis the rotor rotates about, the theoretical results are the same. Here, we consider a γ counter-clockwise rotation of the rotor about the Y-axis. One optoelectronic detector is placed along the OY-axis. In this case, the coordinate OXYZ transfers to OX’Y’Z’ as shown in Figure 3a, where the angle between OX and OX’ is equal to γ and represents the azimuth. This means Φ=γ. The latitude circle O_4_ is formed when the beam of the optoelectronic detector is exposed on the spinning rotor. *L* is the distance between the configuration position of the optoelectronic detector with the center of the latitude circle O_4_. From this figure, it can be seen that the rotated black and white strip widths are different on the latitude circle O_4_. To calculate the strip width, we can take the γ counter-clockwise rotation of the rotor about the Y-axis as the γ clockwise rotation of the latitude circle O_4_ about the Y-axis while the rotor keeps its original spinning status. This can be observed in Figure 3b.

The circle O_5_ across Points G, H, and I can be obtained by an α clockwise rotation of the longitudinal circle O_1_ about OX and expressed as

(5) $$\left\{\begin{array}{c} x^{2}+y^{2}+z^{2}=R^{2}\\ -y\sin\alpha +z\cos\alpha =0 \end{array}\right..$$

When the latitude circle O_4_ has a γ clockwise rotation about OY, its equation is obtained as

(6) $$\left\{\begin{array}{c} x^{2}+y^{2}+z^{2}=R^{2}\\ x\cos\gamma +z\sin\gamma =b \end{array}\right.$$

where *b* represents the height of the latitude circle O_4_.

From Figure 3b, one can see that the lengths of the arcs LN and LM present the black and white stripe widths. Thus, the duty ratio is calculated as $K=\frac{\hat{LM}}{\hat{LM}+\hat{LN}}=\frac{\hat{LM}}{\hat{MN}}$. Point L is the intersection between Circles O_2_ and O_4_, so Equations (1) and (6) can obtain the coordinate ($x_{L}$, $y_{L}$, $z_{L}$) of Point L as

(7) $$\left\{\begin{array}{c} x_{L}=b\cos\beta \sec\left(\beta +\gamma \right)\\ y_{L}=\sqrt{R^{2}-b^{2}\sec^{2}\left(\beta +\gamma \right)}\\ z_{L}=-b\sin\beta \sec\left(\beta +\gamma \right) \end{array}\right..$$

Similarly, Point M is the intersection between Circles O_3_ and O_4_, so Equations (2) and (6) can yield

(8) $$\left\{\begin{array}{c} x_{M}=\frac{b\cos\gamma +\sin\alpha \sin\gamma \sqrt{R^{2}\left(\cos^{2}\alpha \cos^{2}\gamma +\sin^{2}\alpha \right)-b^{2}}}{\cos^{2}\alpha \cos^{2}\gamma +\sin^{2}\alpha }\\ y_{M}=\frac{-b\sin\alpha \cos\alpha \sin\gamma +\cos\alpha \cos\gamma \sqrt{R^{2}\left(\cos^{2}\alpha \cos^{2}\gamma +\sin^{2}\alpha \right)-b^{2}}}{\cos^{2}\alpha \cos^{2}\gamma +\sin^{2}\alpha }\\ z_{M}=\frac{b\sin\gamma \sin^{2}\alpha -\sin\alpha \cos\gamma \sqrt{R^{2}\left(\cos^{2}\alpha \cos^{2}\gamma +\sin^{2}\alpha \right)-b^{2}}}{\cos^{2}\alpha \cos^{2}\gamma +\sin^{2}\alpha } \end{array}\right..$$

According to Equations (5) and (6), the common point N between Circles O_4_ and O_5_ equals

(9) $$\left\{\begin{array}{c} x_{N}=\frac{b\cos\gamma -\sin\alpha \sin\gamma \sqrt{R^{2}\left(\cos^{2}\alpha \cos^{2}\gamma +\sin^{2}\alpha \right)-b^{2}}}{\cos^{2}\alpha \cos^{2}\gamma +\sin^{2}\alpha }\\ y_{N}=\frac{b\sin\alpha \cos\alpha \sin\gamma +\cos\alpha \cos\gamma \sqrt{R^{2}\left(\cos^{2}\alpha \cos^{2}\gamma +\sin^{2}\alpha \right)-b^{2}}}{\cos^{2}\alpha \cos^{2}\gamma +\sin^{2}\alpha }\\ z_{N}=\frac{b\sin\gamma \sin^{2}\alpha +\sin\alpha \cos\gamma \sqrt{R^{2}\left(\cos^{2}\alpha \cos^{2}\gamma +\sin^{2}\alpha \right)-b^{2}}}{\cos^{2}\alpha \cos^{2}\gamma +\sin^{2}\alpha } \end{array}\right..$$

Based on Equations (7) and (8), the length of the arc LM equals

(10) $$\begin{array}{cc} \hat{LM} & =R\arccos\frac{2R^{2}-\left[{\left(x_{L}-x_{M}\right)}^{2}+{\left(y_{L}-y_{M}\right)}^{2}+{\left(z_{L}-Z_{M}\right)}^{2}\right]}{2R^{2}} \end{array}.$$

Using Equations (8) and (9), the length of the arc MN is calculated as

(11) $$\begin{array}{cc} \hat{MN} & =R\arccos\frac{2R^{2}-\left[{\left(x_{N}-x_{M}\right)}^{2}+{\left(y_{N}-y_{M}\right)}^{2}+{\left(z_{N}-Z_{M}\right)}^{2}\right]}{2R^{2}} \end{array}.$$

From Equations (10) and (11), we then obtain the duty ratio $K=\frac{\hat{LM}}{\hat{MN}}$. When b=0, the duty ratio $K=\frac{1}{2}$ constantly and is unrelated with the azimuth Φ. Thus, in real implementation, the optoelectronic detector may be placed in any latitude circle except the equinoctial circle O*_E_*.

## 4. Two-Dimensional Rotation

**Figure 4:**
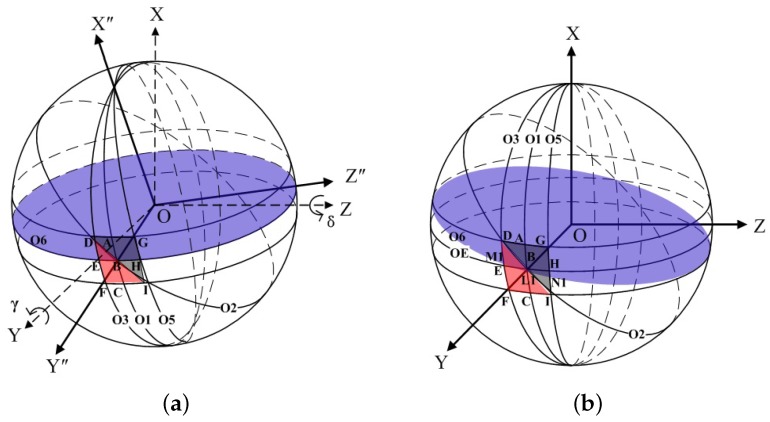
(**a**) Rotation of OXYZ about OY and OZ. (**b**) Rotation of Circle O_6_ about OY and OZ.

Here, we denote a γ counter-clockwise rotation of the rotor about OY, followed by a δ counter-clockwise rotation about OZ. The frame OXYZ then produces a new orientation OX”Y”Z” as shown in Figure 4a. The γ angle orientation is a description of the pitch attitude of the object, and the δ angle orientation depicts the yaw attitude of the object. In the new orientation OX”Y”Z”, the unit vector of the OX-axis (1,0,0) in the original OXYZ frame is transformed to (cosγcosδ,cosγsinδ,−sinγ). Thus, the angle between OX and OX” represents the azimuth Φ=arccos(cosγcosδ), which indicates that the γ and δ angles have to be known. To do this, two optoelectronic detectors are required and configured along the OY- and OZ-axes, resulting in two duty ratio signals $K_{1}$ and $K_{2}$. They are related to γ and δ angles, which are to be introduced in the following.

### 4.1. Duty Ratio $K_{1}$

Similar to one-dimensional rotation, the duty ratio $K_{1}$ is determined according to the different black and white stripe widths, i.e., $K=\frac{\hat{L_{1}M_{1}}}{\hat{L_{1}M_{1}}+\hat{L_{1}N_{1}}}=\frac{\hat{L_{1}M_{1}}}{\hat{M_{1}N_{1}}}$. This can be observed in Figure 4b. To calculate them, the two-dimensional rotation of the rotor described above is equivalently regarded as a γ clockwise rotation of the latitude circle O_6_ about OY, followed by a δ clockwise rotation of O_6_ about OZ. At the same time, the rotor remains unchanged. The circle O_6_ after the rotation is expressed as

(12) $$\left\{\begin{array}{c} x^{2}+y^{2}+z^{2}=R^{2}\\ x\cos\gamma \cos\delta -y\cos\gamma \sin\delta +z\sin\gamma =b \end{array}\right..$$

From Figure 4b, Point L_1_ is the intersection between Circles O_2_ and O_6_, so Equations (1) and (12) can obtain the coordinate ($x_{L_{1}}$, $y_{L_{1}}$, $z_{L_{1}}$) of Point L_1_ as

(13) $$\left\{\begin{array}{c} x_{L_{1}}=\frac{b\cos\beta \left(\cos\beta \cos\gamma \cos\delta -\sin\beta \sin\gamma \right)+\cos\beta \cos\gamma \sin\delta {\mathrm{TempL}}_{1}}{1-{\left(\cos\beta \sin\gamma +\sin\beta \cos\gamma \sin\delta \right)}^{2}}\\ y_{L_{1}}=\frac{-b\cos\gamma \sin\delta +\left(\cos\beta \cos\gamma \cos\delta -\sin\beta \sin\gamma \right){\mathrm{TempL}}_{1}}{1-{\left(\cos\beta \sin\gamma +\sin\beta \cos\gamma \sin\delta \right)}^{2}}\\ z_{L_{1}}=\frac{-b\sin\beta \left(\cos\beta \cos\gamma \cos\delta -\sin\beta \sin\gamma \right)-\sin\beta \sin\delta \cos\gamma {\mathrm{TempL}}_{1}}{1-{\left(\cos\beta \sin\gamma +\sin\beta \cos\gamma \sin\delta \right)}^{2}} \end{array}\right.$$

where ${\mathrm{TempL}}_{1}=\sqrt{-b^{2}+R^{2}\left[1-{\left(\cos\beta \sin\gamma +\sin\beta \cos\gamma \sin\delta \right)}^{2}\right]}$.

Point M_1_ is the intersection between Circles O_3_ and O_6_, and Equations (2) and (12) can yield

(14) $$\left\{\begin{array}{c} x_{M_{1}}=\frac{b\cos\gamma \cos\delta +\left(\cos\alpha \cos\gamma \sin\delta +\sin\alpha \sin\gamma \right){\mathrm{TempM}}_{1}}{1-{\left(\cos\alpha \sin\gamma -\sin\alpha \cos\gamma \sin\delta \right)}^{2}}\\ y_{M_{1}}=\frac{-b\cos\alpha \left(\sin\alpha \sin\gamma +\cos\alpha \cos\gamma \sin\delta \right)+\cos\alpha \cos\gamma \cos\delta {\mathrm{TempM}}_{1}}{1-{\left(\cos\alpha \sin\gamma -\sin\alpha \cos\gamma \sin\delta \right)}^{2}}\\ z_{M_{1}}=\frac{b\sin\alpha \left(\sin\alpha \sin\gamma +\cos\alpha \cos\gamma \sin\delta \right)-\sin\alpha \cos\gamma \cos\delta {\mathrm{TempM}}_{1}}{1-{\left(\cos\alpha \sin\gamma -\sin\alpha \cos\gamma \sin\delta \right)}^{2}} \end{array}\right.$$

where ${\mathrm{TempM}}_{1}=\sqrt{-b^{2}+R^{2}\left[1-{\left(\cos\alpha \sin\gamma -\sin\alpha \cos\gamma \sin\delta \right)}^{2}\right]}$.

Based on Equations (5) and (12), the intersection N_1_ between Circles O_5_ and O_6_ equals

(15) $$\left\{\begin{array}{c} x_{N_{1}}=\frac{b\cos\gamma \cos\delta -\left(\sin\alpha \sin\gamma -\cos\alpha \cos\gamma \sin\delta \right){\mathrm{TempN}}_{1}}{1-{\left(\cos\alpha \sin\gamma +\sin\alpha \cos\gamma \sin\delta \right)}^{2}}\\ y_{N_{1}}=\frac{b\cos\alpha \left(\sin\alpha \sin\gamma -\cos\alpha \cos\gamma \sin\delta \right)+\cos\alpha \cos\gamma \cos\delta {\mathrm{TempN}}_{1}}{1-{\left(\cos\alpha \sin\gamma +\sin\alpha \cos\gamma \sin\delta \right)}^{2}}\\ z_{N_{1}}=\frac{b\sin\alpha \left(\sin\alpha \sin\gamma -\cos\alpha \cos\gamma \sin\delta \right)+\sin\alpha \cos\gamma \cos\delta {\mathrm{TempN}}_{1}}{1-{\left(\cos\alpha \sin\gamma +\sin\alpha \cos\gamma \sin\delta \right)}^{2}} \end{array}\right.$$

where ${\mathrm{TempN}}_{1}=\sqrt{-b^{2}+R^{2}\left[1-{\left(\cos\alpha \sin\gamma +\sin\alpha \cos\gamma \sin\delta \right)}^{2}\right]}$.

According to Equations (13) and (14), the length of the arc L_1_M_1_ equals

(16) $$\begin{array}{cc} \hat{L_{1}M_{1}} & =R\arccos\frac{2R^{2}-\left[{\left(x_{L_{1}}-x_{M_{1}}\right)}^{2}+{\left(y_{L_{1}}-y_{M_{1}}\right)}^{2}+{\left(z_{L_{1}}-Z_{M_{1}}\right)}^{2}\right]}{2R^{2}} \end{array}.$$

Using Equations (14) and (15), the length of the arc M_1_N_1_ is calculated as

(17) $$\begin{array}{cc} \hat{M_{1}N_{1}} & =R\arccos\frac{2R^{2}-\left[{\left(x_{N_{1}}-x_{M_{1}}\right)}^{2}+{\left(y_{N_{1}}-y_{M_{1}}\right)}^{2}+{\left(z_{N_{1}}-z_{M_{1}}\right)}^{2}\right]}{2R^{2}} \end{array}.$$

Then, from Equations (16) and (17), the duty ratio $K_{1}$ is obtained, i.e., $K_{1}=\frac{\hat{L_{1}M_{1}}}{\hat{M_{1}N_{1}}}$.

### 4.2. Duty Ratio $K_{2}$

**Figure 5:**
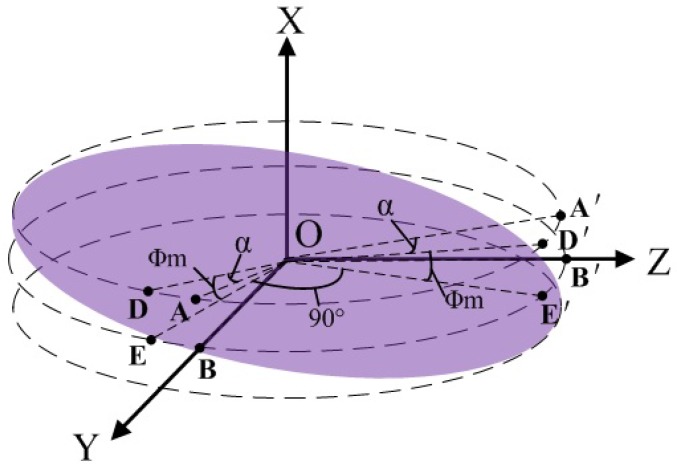
Varied positions of some of the points on O_2_, O_3_, and O_5_.

Similar to the duty ratio $K_{1}$, $K_{2}$ is determined according to the widths of the white and black stripes that are detected by the optoelectronic detector installed in the OZ-axis. It is likely that the stripe width difference is caused by the γ and δ counter-clockwise rotation of the rotor about OY and about OZ, respectively. In this case, the latitude circle O_6_ including the rotor remains unchanged and is expressed by Equation (12). However, the position of the circles O_2_, O_3_, and O_5_ is changed. Figure 5 illustrates the varied position of some of the points on these circles.

The circle O_2_ across the arc D’B’I’ is obtained by a β clockwise rotation of the circle O_1_ about OY and expressed as

(18) $$\left\{\begin{array}{c} x^{2}+y^{2}+z^{2}=R^{2}\\ -x\sin\beta +y\cos\beta =0 \end{array}\right..$$

The circle O_3_ across points D’, E’, and F’ is obtained by a α+90 counter-clockwise rotation of the circle O_1_ about OX and expressed as

(19) $$\left\{\begin{array}{c} x^{2}+y^{2}+z^{2}=R^{2}\\ y\cos\alpha -z\sin\alpha =0 \end{array}\right..$$

The circle O_5_ across points G’, H’, and I’ is obtained by a α+90 clockwise rotation of the circle O_1_ about OX and expressed as

(20) $$\left\{\begin{array}{c} x^{2}+y^{2}+z^{2}=R^{2}\\ y\cos\alpha +z\sin\alpha =0 \end{array}\right..$$

Furthermore, we can obtain the intersection points L_2_ between circles O_2_ and O_6_, M_2_ between circles O_3_ and O_6_, and N_2_ between circles O_5_ and O_6_. They are given as follows:

(21) $$\left\{\begin{array}{c} x_{L_{2}}=\frac{b\cos\beta \cos\gamma \cos\left(\beta +\delta \right)-\cos\beta \sin\gamma {\mathrm{TempL}}_{2}}{1-\sin^{2}\left(\beta +\delta \right)\cos^{2}\gamma }\\ y_{L_{2}}=\frac{b\sin\beta \cos\gamma \cos\left(\beta +\delta \right)-\sin\beta \sin\gamma {\mathrm{TempL}}_{2}}{1-\sin^{2}\left(\beta +\delta \right)\cos^{2}\gamma }\\ z_{L_{2}}=\frac{b\sin\gamma +\cos\gamma \cos\left(\beta +\gamma \right){\mathrm{TempL}}_{2}}{1-\sin^{2}\left(\beta +\delta \right)\cos^{2}\gamma } \end{array}\right.$$

where ${\mathrm{TempL}}_{2}=\sqrt{-b^{2}+R^{2}\left[1-\sin^{2}\left(\beta +\delta \right)\cos^{2}\gamma \right]}$.

(22) $$\left\{\begin{array}{c} x_{M_{2}}=\frac{b\cos\gamma \cos\delta +\left(\sin\alpha \sin\delta \cos\gamma -\cos\alpha \sin\gamma \right){\mathrm{TempM}}_{2}}{1-{\left(\sin\alpha \sin\gamma +\cos\alpha \cos\gamma \sin\delta \right)}^{2}}\\ y_{M_{2}}=\frac{b\sin\alpha \left(\cos\alpha \sin\gamma -\sin\alpha \cos\gamma \sin\delta \right)+\sin\alpha \cos\gamma \cos\delta {\mathrm{TempM}}_{2}}{1-{\left(\sin\alpha \sin\gamma +\cos\alpha \cos\gamma \sin\delta \right)}^{2}}\\ z_{M_{2}}=\frac{b\cos\alpha \left(\cos\alpha \sin\gamma -\sin\alpha \cos\gamma \sin\delta \right)+\cos\alpha \cos\gamma \cos\delta {\mathrm{TempM}}_{2}}{1-{\left(\sin\alpha \sin\gamma +\cos\alpha \cos\gamma \sin\delta \right)}^{2}} \end{array}\right.$$

where ${\mathrm{TempM}}_{2}=\sqrt{-b^{2}+R^{2}\left[1-{\left(\sin\alpha \sin\gamma +\cos\alpha \cos\gamma \sin\delta \right)}^{2}\right]}$.

(23) $$\left\{\begin{array}{c} x_{N_{2}}=\frac{b\cos\gamma \cos\delta -\left(\cos\alpha \sin\gamma +\sin\alpha \cos\gamma \sin\delta \right){\mathrm{TempN}}_{2}}{1-{\left(\sin\alpha \sin\gamma -\cos\alpha \cos\gamma \sin\delta \right)}^{2}}\\ y_{N_{2}}=\frac{b\sin\alpha \left(\cos\alpha \sin\gamma +\sin\alpha \cos\gamma \sin\delta \right)+\sin\alpha \cos\gamma \cos\delta {\mathrm{TempN}}_{2}}{1-{\left(\sin\alpha \sin\gamma -\cos\alpha \cos\gamma \sin\delta \right)}^{2}}\\ z_{N_{2}}=\frac{b\cos\alpha \left(\cos\alpha \sin\gamma +\sin\alpha \cos\gamma \sin\delta \right)+\cos\alpha \cos\gamma \cos\delta {\mathrm{TempN}}_{2}}{1-{\left(\sin\alpha \sin\gamma -\cos\alpha \cos\gamma \sin\delta \right)}^{2}} \end{array}\right.$$

where ${\mathrm{TempN}}_{2}=\sqrt{-b^{2}+R^{2}\left[1-{\left(\sin\alpha \sin\gamma -\cos\alpha \cos\gamma \sin\delta \right)}^{2}\right]}$.

According to the distance between the spherical points, we can further achieve the duty ratio $K_{2}=\frac{\hat{L_{2}M_{2}}}{\hat{M_{2}N_{2}}}$.

## 5. Numerical Solution

In the proposed seeker-azimuth determination approach, the duty ratios $K_{1}$ and $K_{2}$ can be measured directly, but the azimuth Φ needs to be determined from the information of γ and δ according to Φ=arccos(cosγcosδ). In this case, we first need to determine how γ and δ are related to $K_{1}$ and $K_{2}$ so that γ and δ are calculated accordingly. Based on this, the azimuth Φ is determined. From what we have described above, it can be found that the manner in which γ and δ is related to $K_{1}$ and $K_{2}$ is nonlinear. Here, an approximated method is adopted to obtain their relationship. To suit the real requirement, the system parameters are selected as $R=1,\alpha =\frac{\pi }{16},\beta =\frac{\pi }{9}$. The range of γ and δ are set in the range [−0.4rad,0.4rad], respectively. At an interval of 0.01 rad, the γ and δ data points are chosen in this range and input into Equations (13)–(17). We then obtain a set of $K_{1}$ data points. Similarly, the same γ and δ data points with an interval of 0.01 rad are input into Equations (21)–(23), and a set of $K_{2}$ data points are obtained. Based on these data points, the polynomial functions [19] are applied to approximate the mathematical equations depicting $K_{1}=f_{1}\left(\gamma ,\beta \right)$ and $K_{2}=f_{2}\left(\gamma ,\beta \right)$, which are given as

(24) $$\begin{array}{cc} K_{1} & =f_{1}\left(\gamma ,\beta \right)\\ & =0.5-0.0000001517\gamma -0.9268\delta +0.0000002943\gamma^{2}-0.435\gamma \delta -0.0000003576\delta^{2}\\ & -0.00000005382\gamma^{3}-0.1361\gamma^{2}\delta +0.000005755\gamma \delta^{2}-0.2551\delta^{3}-0.000002704\gamma^{4}\\ & -0.1943\gamma^{3}\delta +0.000002178\gamma^{2}\delta^{2}+0.1297\gamma \delta^{3}+0.000002338\delta^{4}+0.00001957\gamma^{5}\\ & -0.1035\gamma^{4}\delta -0.0001071\gamma^{3}\delta^{2}+0.2169\gamma^{2}\delta^{3}+0.00004104\gamma \delta^{4}-0.1433\delta^{5} \end{array}$$

(25) $$\begin{array}{cc} K_{2} & =f_{2}\left(\gamma ,\beta \right)\\ & =0.5+0.9268\gamma +0.00007863\delta +0.3378\gamma^{2}+0.09762\gamma \delta -0.0009562\delta^{2}+0.8426\gamma^{3}\\ & +0.9197\gamma^{2}\delta -0.9053\gamma \delta^{2}-0.002693\delta^{3}+0.8832\gamma^{4}-0.1119\gamma^{3}\delta +0.5357\gamma^{2}\delta^{2}\\ & -1.253\gamma \delta^{3}+0.01843\delta^{4}+1.235\gamma^{5}-0.2093\gamma^{4}\delta -3.026\gamma^{3}\delta^{2}+2.093\gamma^{2}\delta^{3}\\ & +0.1357\gamma \delta^{4}+0.00593\delta^{5}+0.1227\gamma^{6}-2.626\gamma^{5}\delta +2.524\gamma^{4}\delta^{2}-1.05\gamma^{3}\delta^{3}\\ & -0.5787\gamma^{2}\delta^{4}+1.023\gamma \delta^{5}-0.02855\delta^{6}-1.845\gamma^{7}+2.141\gamma^{6}\delta -3.503\gamma^{5}\delta^{2}\\ & -0.04513\gamma^{4}\delta^{3}+8.729\gamma^{3}\delta^{4}-6.163\gamma^{2}\delta^{5}+0.2892\gamma \delta^{6}+0.1944\delta^{7}-0.6482\gamma^{8}\\ & -1.973\gamma^{7}\delta +1.631\gamma^{6}\delta^{2}+14.2\gamma^{5}\delta^{3}-19.76\gamma^{4}\delta^{4}+2.907\gamma^{3}\delta^{5}+6.077\gamma^{2}\delta^{6}\\ & -1.852\gamma \delta^{7}-0.4197\delta^{8}-3.77\gamma^{9}+5.774\gamma \gamma^{8}\delta +9.071\gamma^{7}\delta^{2}-30.43\gamma^{6}\delta^{3}+25.65\gamma^{5}\delta^{4}\\ & +9.478\gamma^{4}\delta^{5}-24.79\gamma \gamma^{3}\delta^{6}+7.587\gamma^{2}\delta^{7}+2.981\gamma \delta^{8}-0.83\delta^{9} \end{array}.$$

In a similar method, we determine the mathematical equations $\gamma =f_{3}\left(K_{1},K_{2}\right)$ and $\delta =f_{4}\left(K_{1},K_{2}\right)$. The range of duty ratios $K_{1}$ and $K_{2}$ are set in the range of [0.25,0.85]. Then, with an interval of 0.01, a set of $K_{1}$ and $K_{2}$ data points are selected. With zero initial values of γ, δ, $K_{1}$, and $K_{2}$ data points, the Newton numerical technique [20] is applied to solve Equations (24) and (25). In this case, the mathematical equations $\gamma =f_{3}\left(K_{1},K_{2}\right)$, $\delta =f_{4}\left(K_{1},K_{2}\right)$ are gained. They are expressed as follows:

(26) $$\begin{array}{cc} \gamma & =f_{3}\left(K_{1},K_{2}\right)\\ & =-3.585+13.49K_{1}+14.64K_{2}-24.34K_{1}^{2}-45.7K_{1}K_{2}-24.71K_{2}^{2}+22.51K_{1}^{3}+58.8K_{1}^{2}K_{2}\\ & +57.35K_{1}K_{2}^{2}+24.64K_{2}^{3}-11.04K_{1}^{4}-33.21K_{1}^{3}K_{2}-48.53K_{1}^{2}K_{2}^{2}-30.88K_{1}K_{2}^{3}-14.69K_{2}^{4}\\ & +2.501K_{1}^{5}+6.175K_{1}^{4}K_{2}+15.14K_{1}^{3}K_{2}^{2}+11.88K_{1}^{2}K_{2}^{3}+6.265K_{1}K_{2}^{4}+4.069K_{2}^{5} \end{array}$$

(27) $$\begin{array}{cc} \delta & =f_{4}\left(K_{1},K_{2}\right)\\ & =-0.05233-0.1852K_{1}+0.004281K_{2}+0.00113K_{2}^{3}+0.01551K_{1}K_{2}-0.0008619K_{2}^{2}\\ & +0.002093K_{2}^{3}+0.0008284K_{2}^{2}K_{2}-0.0009574K_{1}K_{2}^{2}+0.0001974K_{2}^{3}+0.0001074K_{1}^{4}\\ & -0.0001137K_{1}^{3}K_{2}+0.0007012K_{1}^{2}K_{2}^{2}+0.000122K_{1}K_{2}^{3}+0.0001014K_{2}^{4}-0.00008928K_{2}^{5}\\ & -0.0002575K_{1}^{4}K_{2}-0.0002451K_{1}^{3}K_{2}^{2}-0.0004166K_{1}^{2}K_{2}^{3}-0.0001407K_{1}K_{2}^{4}-0.00003762K_{2}^{5} \end{array}.$$

On the basis of Equations (26) and (27), the azimuth angle is then computed as $\Phi =\arccos\left(\cos\gamma \cos\delta \right)$.

## 6. Experimental Results

**Figure 6:**
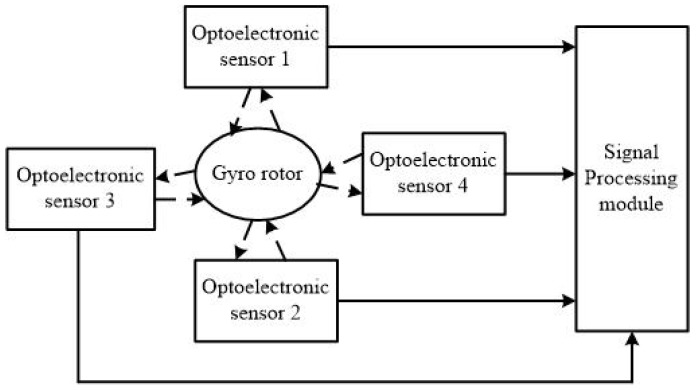
System diagram.

**Figure 7:**
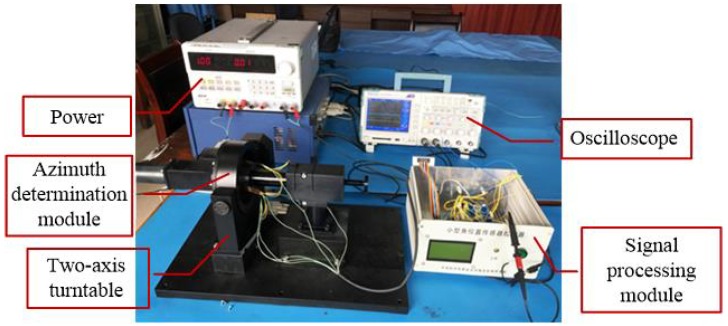
Real experiment platform.

**Figure 8:**
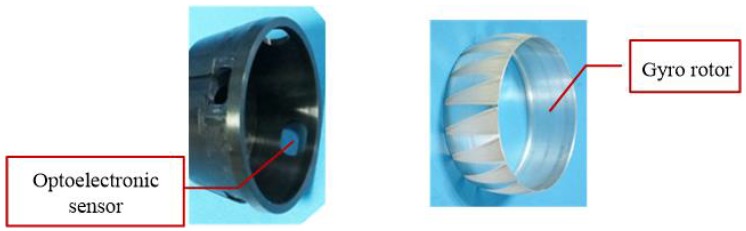
Azimuth determination module.

In this section, the experimental setup used to verify and test the concept is described. The system diagram is shown in Figure 6. The real experiment platform and azimuth determination module are depicted in Figure 7 and Figure 8, respectively. Since two optoelectronic sensors have a limited working range, four optoelectronic sensors are applied to enlarge the working range. Two are installed along the OY-axis in the opposite direction and the other two are installed along the OZ-axis in the opposite direction, as shown in Figure 6. The signal processing module is used to implement data handling and functional operation to calculate the real azimuth according to Equations (26) and (27). The total four duty ratios $K_{i},i=1,\cdots ,4$ are produced accordingly. Because the installation of four optoelectronic sensors satisfies the orthogonal relationship, only two optoelectronic sensors output the duty ratios, while the other two optoelectronic sensors have zero duty ratios. The resulting duty ratios in different rotation types are summarized in Table 1.

**Figure 9:**
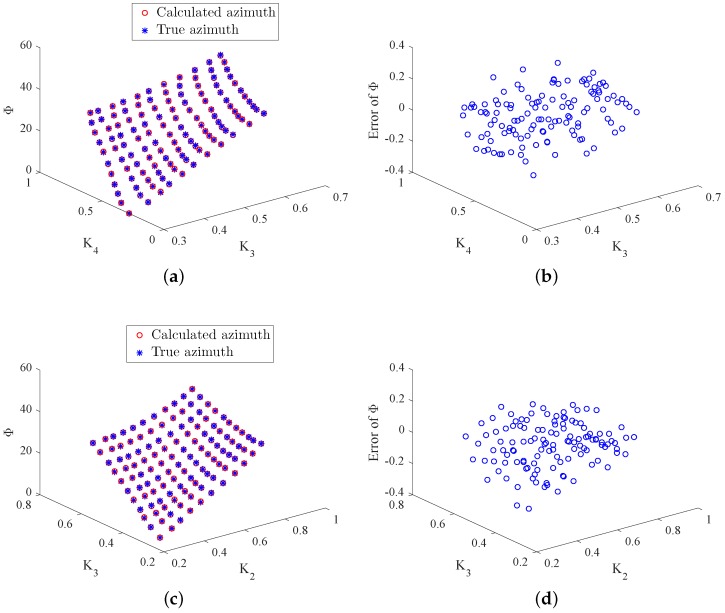
True Φ, calculated Φ, and error of Φ in Working States 1 and 2. (**a**) Φ in State 1; (**b**) error of Φ in State 1; (**c**) Φ in State 2; (**d**) error of Φ in State 2.

**Figure 10:**
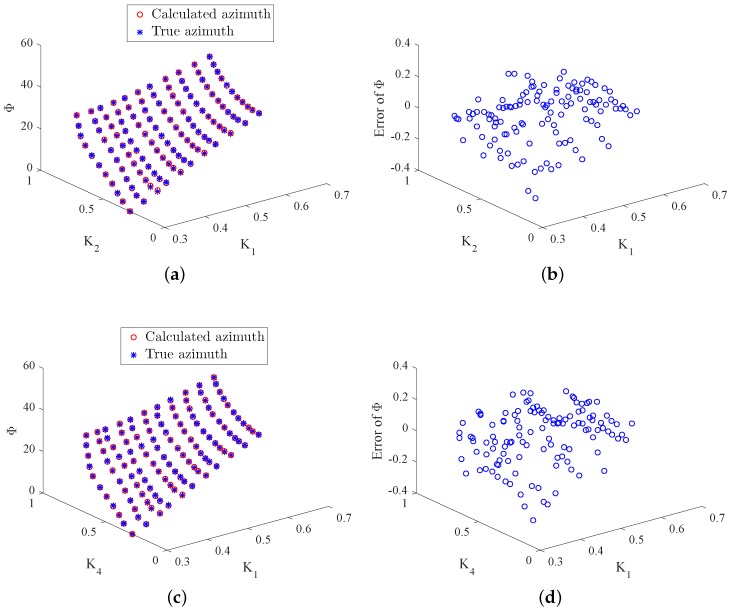
True Φ, calculated Φ, and error of Φ in Working States 3 and 4. (**a**) Φ in State 3; (**b**) error of Φ in State 3; (**c**) Φ in State 4; (**d**) error of Φ in State 4.

**Figure 11:**
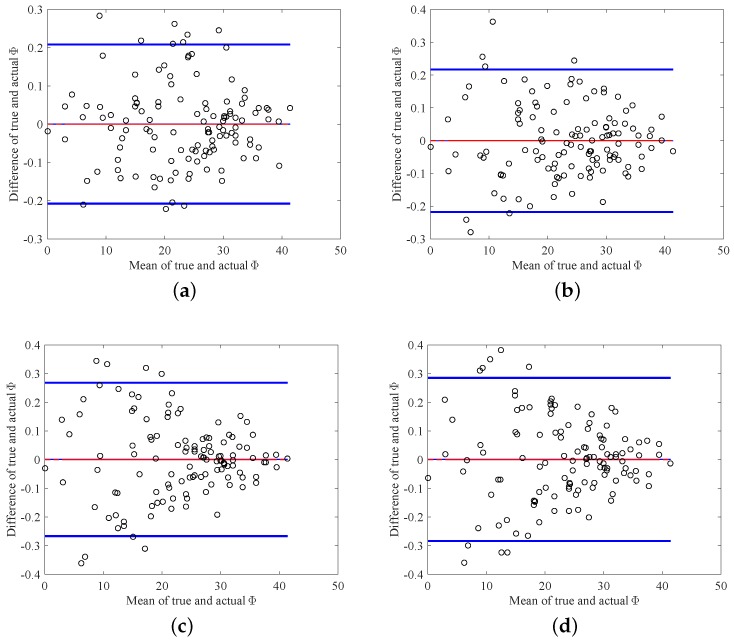
The Bland–Altman plot of true Φ and calculated Φ in different working states. (**a**) State 1; (**b**) State 2; (**c**) State 3; (**d**) State 4.

In real implementation, two duty ratios are measured by the optoelectronic sensors in each working state and then the azimuth angle is calculated on the basis of Equations (26) and (27) given above. To evaluate the effectiveness of the proposed approach, the theoretical Φ values need to be known. In the experiment, the two-axis turntable is applied to generate the theoretical azimuth values. It simulates the theoretical Y-axis and Z-axis rotations for producing the theoretical γ and δ. Then the theoretical azimuth values are obtained through $\Phi =\arccos\left(\cos\gamma \cos\delta \right)$. It is noteworthy that, in practice, the Y-axis and Z-axis rotations are produced by the real system and the duty ratio *K* is then produced. Furthermore, the real azimuth values are achieved through the proposed approach. Figure 9 and Figure 10 depict the true and calculated azimuth angle values for the four working states. From these figures, one can observe that the four duty ratios lie in the range [0.3,0.8], and the range of the azimuth is [0,40]. According to the design requirement, the standard deviation between the true and calculated azimuth angles is required to be less than than 0.005. Table 2 summarizes the standard deviation between the desired and real azimuth values under four working states. From the table, it can be observed that the standard deviations of all the four working states reach the design goal. Additionally, Figure 11a–d gives the Bland–Altman plots, which represent the differences between the true Φ and the measured Φ in the four working states. In these figures, the two blue lines show error bars representing the 95% confidence interval for both the upper and lower limits of agreement. The red line represents the mean of the differences between the true and measured Φ. The black line depicts zero values. From these figures, one can find that almost all of the measure values fall inside the agreement interval, which indicates that the measured Φ and the true Φ have good statistical agreement. Moreover, one can see that the mean of the true and measured Φ is very close to zero. This indicates that the error from the designed system is very low.

**Figure 12:**
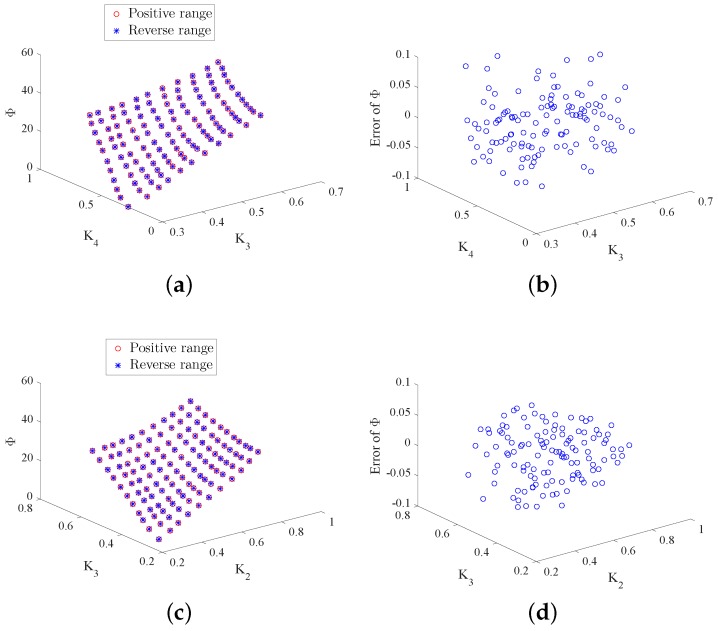
Measured Φ via positive and reverse range and the produced error of Φ in Working States 1 and 2. (**a**) Φ in State 1; (**b**) error of Φ in State 1; (**c**) Φ in State 2; (**d**) error of Φ in State 2.

**Figure 13:**
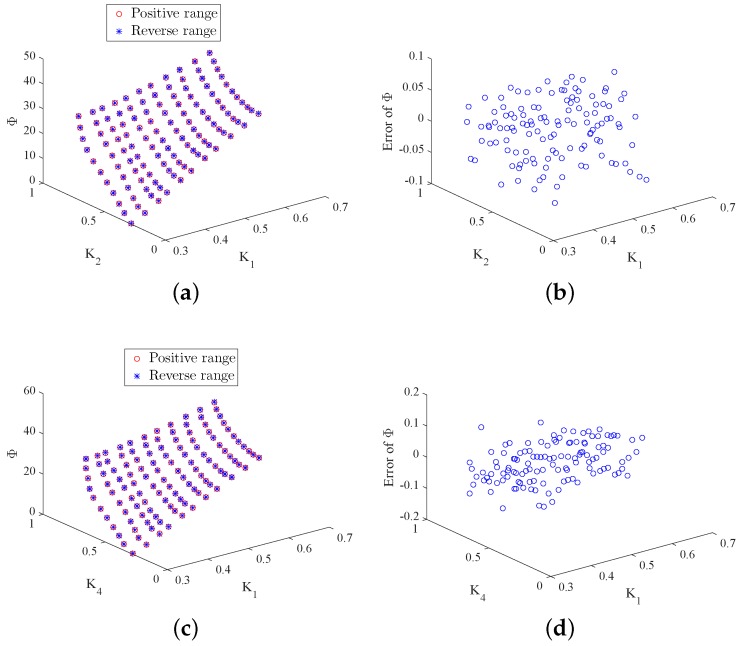
Measured Φ via positive and reverse range and the produced error of Φ in Working States 3 and 4. (**a**) Φ in State 3; (**b**) error of Φ in State 3; (**c**) Φ in State 4; (**d**) error of Φ in State 4.

To further assess the consistency and reproducibility of measurements made by the designed scheme, we conducted three experiments for each working state and adopted the intraclass correlation (ICC) test to analyze the measurement reliability. The ICC results are given in Table 2. From the table, one can note that the ICC results of the four working states are above 0.9, which indicates that the three different experiments are highly correlated. This further verifies the conformity of measurements and the effectiveness of the designed scheme. Figure 12 and Figure 13 show the hysteresis test for the four working states. To achieve this test, we conducted experiments by changing the duty ratios in reverse from the maximum to the minimum. From these figures, we can see that the measurements from the positive range and reverse range match well. The hysteresis errors calculated by the maximum error divided by the maximum measured value are shown in Table 2. It can be found from the table that the hysteresis errors are very small, which further indicates that the hysteresis can be ignored in our designed system.

## 7. Conclusions

In this paper, a new approach for determining the azimuth of the seeker is proposed based on the gyro rotor and optoelectronic sensors. The gyro rotor is comprised of a set of right spherical triangle patterns in a black and white sequence on its surface. The optoelectronic sensors are placed symmetrically around the gyro rotor. The black and white patterns on the gyro rotor are different in case of an azimuth. The duty ratios are then generated when the optoelectronic sensors catch the reflected optical signals on the different patterns. Formulas between the duty ratios and the azimuth are derived when the rotor rotates in a two-dimensional way. To evaluate the effectiveness of the proposed approach, multiple experiments are conducted under four different working states, and the ICC test is utilized to analyze the consistency of measurements. The experimental results show that the proposed approach satisfies the design goal. The ICC results show that different experiments have high correlation, which ensures the conformity of measurements produced by the proposed approach. The Bland–Altman plots between the true Φ and the measured Φ verify that both have good statistical agreement. In addition, the designed system showed little hysteresis. 

## Figures and Tables

**Table 1:** Different working states of the rotor and resulting duty ratios.

Working State	Rotation about OY-Axis	Rotation about OZ-Axis	Duty Ratios
State 1	Counter-clockwise	Counter-clockwise	$K_{3}$, $K_{4}$
State 2	Clockwise	Counter-clockwise	$K_{2}$, $K_{3}$
State 3	Clockwise	Clockwise	$K_{1}$, $K_{2}$
State 4	Counter-clockwise	Clockwise	$K_{1}$, $K_{4}$

**Table 2:** Linearity error, intraclass correlation (ICC), and hysteresis error under four working states.

Working State	State 1	State 2	State 3	State 4
Linearity error	0.00265	0.00276	0.00341	0.00363
ICC under different experiments	0.93070	0.95225	0.96829	0.96147
Hysteresis error	0.00226	0.00177	0.00208	0.00246
